# A central research portal for mining pancreatic clinical and molecular datasets and accessing biobanked samples

**DOI:** 10.1016/j.tranon.2025.102550

**Published:** 2025-10-03

**Authors:** Jorge Oscanoa, Helen Ross-Adams, Abu Z.M. Dayem Ullah, Trupti S. Kolvekar, Lavanya Sivapalan, Emanuela Gadaleta, Graeme J. Thorn, Maryam Abdollahyan, Ahmet Imrali, Amina Saad, Rhiannon Roberts, Christine S. Hughes, PCRFTB, Hemant M. Kocher, Claude Chelala

**Affiliations:** a Centre for Cancer Biomarkers and Biotherapeutics, Barts Cancer Institute, Queen Mary University London, EC1M 6BQ, UK; b Pancreatic Cancer Research Fund Tissue Bank, Centre for Tumour Biology, Barts Cancer Institute, Queen Mary University London, EC1M 6BQ, UK; c Centre for Tumour Biology, Barts Cancer Institute, Queen Mary University London, EC1M 6BQ, UK; d Barts and the London HPB Centre, The Royal London Hospital, Barts Health NHS Trust, Whitechapel, London E1 1BB, UK

**Keywords:** Genomics, Transcriptomics, Translational, Pancreas biobank, Biomarkers

## Abstract

**Objectives:**

We present Pancreas Genome Phenome Atlas (PGPA) as a resource for the mining and analysis of pancreatic -omics datasets, and demonstrate the biological interpretations possible due to this dynamic analytics hub accommodating an extensive range of publicly available datasets.

**Methods:**

Clinical and molecular datasets from four primary sources are included (The Cancer Genome Atlas, International Cancer Genome Consortium, Cancer Cell Line Encyclopaedia, Genomics Evidence Neoplasia Information Exchange), which form the foundation of -omics profiling of pancreatic malignancies and related lesions (*n* = 7760 specimens). Several user-friendly analytical tools to integrate and explore molecular data derived from these primary specimens and cell lines are available. Crucially, PGPA is positioned as the data access point for Pancreatic Cancer Research Fund Tissue Bank – the only national pancreatic cancer biobank in the UK. This will pioneer a new era of biobanking to promote collaborative studies and effective sharing of multi-modal molecular, histopathology and imaging data (>125 000 specimens from >3980 cases and controls; >2700 radiology images, and >2630 digitised H&Es from 401 donors) to accelerate validation of *in silico* findings in patient-derived material.

**Results:**

We demonstrate the practical utility of PGPA by investigating somatic variants associated with established transcriptomic subtypes and disease prognosis: several patient-specific variants are clinically actionable and may be leveraged for precision medicine.

**Conclusions:**

This places PGPA at the analytical forefront of pancreatic biomarker-based research, providing the user community with a distinct resource to facilitate hypothesis-testing on public data, validate novel research findings, and access curated, high-quality patient tissues for translational research.

## Highlights

• PGPA is a central hub for mining and analysing pancreatic -omics datasets.
• It links to the UK’s national Biobank with >125,000 biospecimens & multi-modal data.
• Enables subtype-specific analysis and discovery of actionable mutations.
• Supports data return and sample access to advance translational research.

## Graphical abstract

**Figure:**
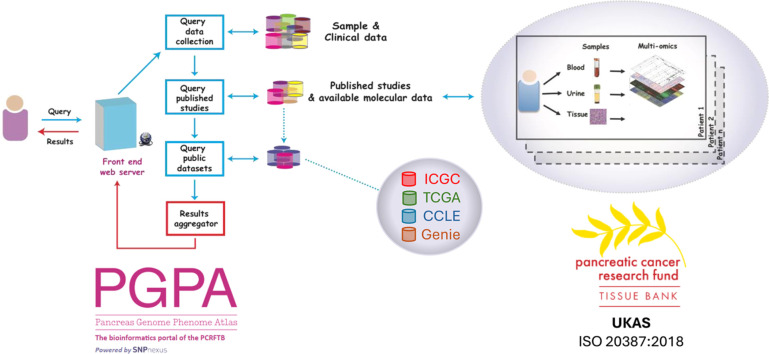

## Introduction

Pancreatic ductal adenocarcinoma (PDAC) is predicted to become the second leading cause of cancer-related mortality worldwide before 2040[1]. It has dismal 5-year survival rates of 3–15 %[1,2], largely due to late disease detection and few effective treatment options. Alarmingly, the incidence of early-onset pancreatic cancer is also increasing, in contrast to most other solid tumours[3]. Better tools for patient stratification and treatment response are thus essential to improve survival outcomes. However, the pancreatic cancer research community is relatively small – investigators tend to develop bespoke collections of samples that may be unusable beyond the breadth and scope of their ethical approval and storage conditions. Sample collection protocols also vary widely, further limiting the translation of derived results to clinical benefits.

Most existing biomarkers for monitoring treatment or assessing prognosis are not based on the molecular attributes of PDAC tumours and have shown limited sensitivity and/or specificity in prospective settings[4]. Numerous studies into the genomic and transcriptomic determinants of tumour development and progression are available, but their findings are dispersed across multiple resources and can be difficult to access and translate into meaningful survival or treatment benefits for patients by those without computational expertise. This highlights the pressing need for simplified, integrated data mining and analysis tools to improve accessibility to clinical and molecular information from disparate sources and enable laboratory and clinical researchers to easily and effectively cross-query large multi-omics datasets, to fuel new discoveries in pancreatic diseases.

Here, we present the Pancreas Genome Phenome Atlas (PGPA), an intuitive, online portal that links numerous multi-modal datasets to an active biobank where users can both validate their findings, and/or apply for samples to confirm *in silico* findings. This represents a significant expansion in functionality from our old 2018 Pancreas Expression Database [5] (now retired): more analytic tools, expanded datasets to encompass the broad spectrum of pancreatic lesions enriched with multi-modal data (demographic, clinical, transcriptomic and genomic) and links to an active biobank. This website is free and open to all users and there is no login requirement.

To remain abreast of the evolving nature of integrative multi-omics workflows, we have included a broad range of available datasets and placed this essential resource at the centre of an established framework (Table 1). PGPA is also integrated as the major bioinformatics platform of the UK’s national Pancreatic Cancer Research Fund Tissue Bank (PCRFTB), to facilitate investigative biomarker-based research and data sharing between clinicians and scientists. This is powered by a customised version of SNPNexus, a versatile platform for the functional annotation of known and novel sequence variation[6], designed to reduce the analytical burden associated with large-scale genomic datasets and facilitate the straightforward identification of biologically and clinically relevant genetic variants in patients.

**Table 1:** Summary of the features in PGPA.

**Features**	**PED (archived)** [5]	**PGPA**
**The Analytics Hub**		
**Publicly available data sources (pancreas-specific)**		
PubMed^a^	X	
TCGA^b^	X	X
ICGC		X
GENIE^c^	X	X
CCLE^d^	X	X
**Analytical features**		
Principal components analysis	X	X
Gene expression profiles	X	X
Correlation analyses	X	X
Gene networks	X	X
Survival analyses^e^	X	X
Variant identification	X	X
Somatic gene interactions	X	X
Reactome & oncogenic pathway analyses		X
Improved clinical annotations to visualize & query publicly available datasets		X
MAFtools genomic analyses and summary visualisations		X
Tumour mutational burden		X
Clinically actionable genes/proteins & associated drugs		X
Cohort comparison by clinical/molecular feature		X
Gene intersections between filtered datasets		X

**The PCRFTB Data Module**		
Data return module to host both -omics and experimental datasets		X
Integrated primary/secondary care clinical data		X
Apply for samples		X

a Less-used literature mining module.

b Includes essential filters based on cancer subtype.

c Includes full set of 6633 pancreatic cancers of all types, with additional clinical and molecular information.

d Somatic variant dataset; includes the full set of 60 primary and metastatic tumour derived cell lines.

e Includes analyses based on mutational status and mRNA level.

**Fig. 1:**
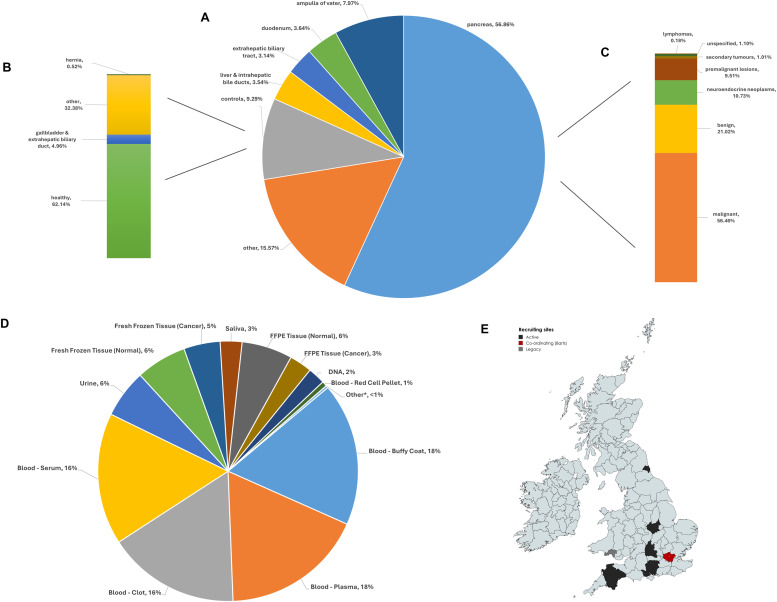
**Summary of available tissue types.** (**A**) The proportion of >3 400 unique organ site tissues available for study in the UK national Pancreatic Cancer Research Fund Tissue Bank, with the breakdown of controls (**B**) and pancreas (**C**) highlighted. (**D**) Distribution of >125 000 PCRFTB specimens by type, across all >3980 patients. Details are updated weekly. Additionally, radiological imaging is available for 2702 patients with malignant, pre-malignant and benign pancreatic diagnoses, and >2630 digitised H&E images from 401 donors. Samples can be applied for here. (**E**) Geographical locations of PCRFTB patient recruitment sites. *=pancreatic juice, CTC, bile, organoids, cancer-associated fibroblasts. Map created with mapchart.net.

The PCRFTB is the world’s first national pancreas tissue bank and has been collecting blood, urine, saliva and solid tissue samples from patients recruited at nine participating centres across the UK NHS since 2015, making it a valuable resource for translational research. Tissues are available from patients with pancreatic and hepatobiliary diseases, including resectable and unresectable cancer. Blood, urine and saliva samples from patients, their first-degree relatives and other healthy volunteers are also available, as well as cancer organoids and cancer-associated fibroblasts (Fig. 1). These are accompanied by extensive and verified clinical, histological and imaging data that is continually updated – median 300 data points per visit, with some donors providing longitudinal samples at multiple visits throughout their treatment journey. Best practice and ongoing technical research ensure available biological materials are of high quality, to support reliable and reproducible results[7,8]. In recognition of PCRFTB’s rigorous technical and ethical standards, it has recently achieved ISO 20387:2018 accreditation [9]. In addition to samples, >2.5 M dcm radiological image files are available for 2702 patients with malignant, pre-malignant and benign pancreatic diagnoses, and 2637 H&E images from 401 donors are also currently available.

The PCRFTB is further supported by a Data Return policy to maximise the use of available samples by linking each patient/donor with an enriched ‘digital fingerprint’ encompassing molecular, transcriptomic, proteomic, imaging and longitudinal clinical data. All donors provide written, informed consent, and all samples are collected, processed and stored at each of the participating centres (Barts, Leicester, Swansea, Oxford, Royal Free (London), Southampton, Newcastle, Plymouth, The London Clinic) under one Research Ethics Committee reference (13/SC/0593, renewed 18/SC/0629, renewed 23/SC/0282) and using standardised protocols, quality assurance and quality control policies ensuring consistency across the collection.

Via PGPA, researchers can directly query and apply to PCRFTB for samples, specimens and/or imaging data that match user-determined criteria. A link to the PCRFTB Tissue Request System allows users to submit Expressions of Interest directly to the Tissue Bank using an online application form.

By providing a dynamic hub for the analysis of publicly available pancreatic datasets and ongoing research data generated from biobanked samples, PGPA allows researchers to access a broad range of pancreas-specific molecular information freely and quickly. The flexibility of this hub allows molecular alterations with biological and clinical relevance to be identified and prioritised for downstream validation.

## The analytics hub

The web-based Analytics Hub includes a broad range of pancreas-related, publicly available -omics datasets, together with extensive analytical features and visualisation options (Table 1).

### Publicly available data sources

PGPA analytics hub hosts publicly available clinical and molecular datasets from four core sources: The Cancer Genome Atlas (TCGA)[10] whole exome sequencing (WES) data, with filters for all available pancreatic cancer types; the Cancer Cell Line Encyclopaedia (CCLE)[11], including somatic mutation and mRNA expression data for the complete set of 60 pancreas cell lines from primary and metastatic tumours; the Genomics Evidence Neoplasia Information Exchange (GENIE)[12] v13.0, including simple somatic mutations and clinical data from the *complete* set of 6633 patients with pancreatic cancer of any type; and the now archived complete International Cancer Genome Consortium (ICGC)[13,14] dataset, including whole genome and RNA sequencing data from both adenocarcinoma (PACA-AU; PACA-CA) and neuro/endocrine tissues (PAEN-IT; PAEN-AU). These sources host data generated by both national and international consortia efforts to sequence and analyse cancer genomes and biology, including pancreatic malignancies. Analysed and quality-controlled data files were downloaded from the respective sources and used without further processing. PGPA 2025 uses the most recent data releases, including linked clinical data when available.

### *Advanced filtering of public datasets*

**Fig. 2:**
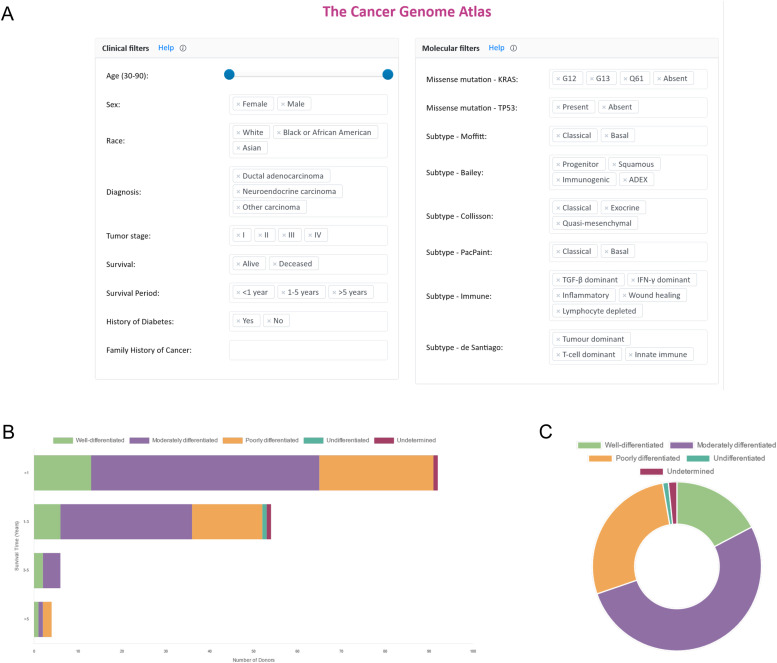
**Advanced filtering options and clinical summaries for publicly available PDAC datasets. (A)** Available data can be filtered according to various patient-related factors and tumour characteristics, including the stratification and analysis of cohorts according to *KRAS* and *TP53* mutational status and established transcriptomic, immune (TCGA, ICGC), genomic (ICGC) or histologically-derived AI (PacPaint) subtypes. **(B)** Dynamic bar charts allow multiple covariates to be viewed in relation to each other: e.g. survival trends in PDAC (TCGA) and neuroendocrine (ICGC PAEN-AU). (**C**) Filtered attributes can also be visualized as summary thumbnail figures for each study cohort; distribution of tumour grade in 185 TCGA PDAC samples is shown as an example. Data underpinning visual outputs can also be downloaded as .csv or .xls files, for offline analysis.

Public datasets may be queried according to the clinical characteristics of each study cohort (Fig. 2A). Filtering options have been selected based on relevance to disease development and pathogenesis, and the depth of annotation provided in available clinical data for each cohort. Implemented filters are accompanied by dedicated visualizations of clinical summaries for each respective study cohort (Fig. 2B). These include filters based on patient-related factors (cancer type, sex, age, diabetes, family history, ethnicity, survival), and tumour characteristics (*KRAS*/*TP53* somatic mutation status, stage, grade (Fig. 2C)), which allow for trends in data to be clearly observed: e.g. survival beyond 3 years is very low for PDAC compared to neuroendocrine tumours (Fig. 2B). Crucially, it is possible to filter each dataset by diagnosis, allowing researchers to focus on different pancreatic lesions individually (e.g. IPMN, ductal adenocarcinoma, neuroendocrine, adenosquamous, mucinous) that have different molecular alterations and clinical prospects, since using unstratified sample sets has been shown to yield unreliable results [15,16].

### *Characterising the genomic characteristics of established PDAC molecular subtypes*

PGPA facilitates the stratification and analysis of TCGA and ICGC cohorts according to their molecular subtype classifications, as determined by hallmark transcriptomic[[17], [18], [19]], genomic (ICGC only)[20] and immunotype [21,22] studies, as well as recent histopathology-based artificial intelligence (AI) predictions in matched TCGA samples[23]. We demonstrate the implementation of this clinically useful feature below.

Collisson et al. (2011) originally identified 3 subclasses of PDAC tumours with different clinical outcomes and treatment responses, termed *quasi-mesenchymal* (QM) (worst prognosis), *classical* (best prognosis) and *exocrine-like*, using hybridisation array-based mRNA expression data from primary untreated resected PDAC[17]. Next, Moffitt et al. (2015) analysed bulk tumour tissues from treatment-naïve primary resected PDAC tumours using virtual microdissection to exclude transcripts native to the normal pancreas and the tumour microenvironment, and reported 2 distinct tumour subtypes (*basal* and *classical*) as well as 2 classifications based on peritumoural stromal tissues (*activated* and *normal*)[18]. *Basal* subtype tumours were associated with a poorer overall patient survival compared to *classical* tumours, which overlap significantly with the Collisson *classical* subtype. Subsequently, Bailey et al. performed RNA-sequencing of bulk primary untreated resected tumour tissues from 328 PDAC tumours and resolved four stable tumour classes (*squamous, pancreatic progenitor, immunogenic* and *aberrantly differentiated endocrine exocrine* (ADEX)), each governed through the differential expression of transcription factors and their targets involved in lineage specification during pancreatic development[19]. *Squamous* subtype tumours overlapped with previous *basal* (Moffitt) and *QM* (Collisson) classifications and were associated with the poorest overall prognosis in patients. Recent investigations of these proposed classifications have corroborated the presence of two overarching transcriptomic subtypes of PDAC tumours comprising *basal-like/squamous* and *classical/progenitor* that have shown relevance for defining survival outcomes in patients, with remaining subtypes (*exocrine-like, ADEX*) shown to have confounding associations with poor tumour cellularity[24,25].

In a complementary approach, an immuno-genomic analysis of >10,000 TCGA tumours across 33 cancer types incorporating data on Th1:Th2-cell ratio; macrophage and/or lymphocyte signatures; level of aneuploidy; neoantigen load; extent of intra-tumoral heterogeneity; rate of cell proliferation; expression of immunomodulatory genes and patient prognosis identified six immune subtypes (*wound healing, IFN-γ dominant, inflammatory, lymphocyte depleted, immunologically quiet* (absent in PDAC)*, TGF-β dominant)* with particular enrichments for cancer driver genes, prognoses and likely therapeutic responses [22]. PDAC were found to be enriched for *inflammatory* subtype, characterised by low/moderate cell proliferation, lower levels of aneuploidy and fewer CNAs, and elevated expression of Th1 and Th2.

Subsequently, de Santiago et al. (2019) performed a meta-analysis of four transcriptomic profiling studies [[17], [18], [19],^26^] to develop a robust immunological classification scheme based on the gene expression signatures from the various tumour-infiltrating immune cell populations in the PDAC microenvironment, using a consensus clustering approach. This identified three distinct immunotypes in PDAC (*adaptive, innate, immune-exclusion*), each with its own prognostic value and potential application in stratifying patients to immunotherapeutic treatments [21].

Most recently, Saillard et al. (2023) used an artificial intelligence model trained and validated on 5 independent surgical and biopsy cohorts with RNAseq and histology data (*n* = 598), including *n* = 126 TCGA samples to further refine these tumour subtypes [23] This approach recapitulated the known *basal/classical* tumour subtypes at the whole H&E slide level but detected variable proportions of basal cells in samples previously categorised as classical subtype when slides were analysed at 112 µm tile size level. This changed survival outcomes in 39 % of cases classed as *classical* subtype by bulk RNAseq analysis, with the impact apparently proportional to the percentage basal cell content [23].

At the DNA level, whole genome sequencing (WGS) and copy number variant (CNV) analysis performed on 100 treatment-naïve, macro-dissected PDAC tissues (ICGC Australia) identified four disease subtypes with distinct patterns of structural variation (*scattered, locally rearranged, stable, unstable)* and clinical utility, with *unstable* subtype characterised by a very high degree of genomic instability throughout the genome and encompassing defects in DNA damage repair (DDR) pathways that confer susceptibility to PARP inhibition (PARPi) and/or platinum chemotherapies[20].

### *Application of subtype-specific filtering criteria in study cohorts*

Subtype-specific characteristics can be explored using TCGA (*n* = 185), ICGC Australia (PACA-AU, *n* = 461) and Canada (PACA-CA, *n* = 317) pancreatic cancer cohorts. Molecular subtype classifications according to Collisson[17], Moffitt[18], Bailey[19], Thorsson [22], de Santiago [21] and/or Saillard[23] are available for *n* ≥ 134 of the 156 confirmed PDAC patients included in the TCGA cohort[24]. Alternatively, ICGC-AU cohorts can be analysed according to the subtype classifications proposed by Bailey et al. (2016)[19] (*n* = 95 patients total; 81 PDAC), de Santiago et al. (2019) (*n* = 204 PDAC)and/or Waddell et al. (2015)(*n* = 86 patients total; 85 PDAC)[20]. Given the prognostic relevance of these transcriptomic subtypes, here we explore the associated genomic features of TCGA PDAC tumours classed unanimously as either classical/progenitor (*n* = 27) or QM/basal-like/squamous (*n* = 16) by all three subtyping systems (Collisson/Moffit/Bailey), as an example of PGPA Analytics Hub.

### *Subtype-specific somatic variations*

**Fig. 3:**
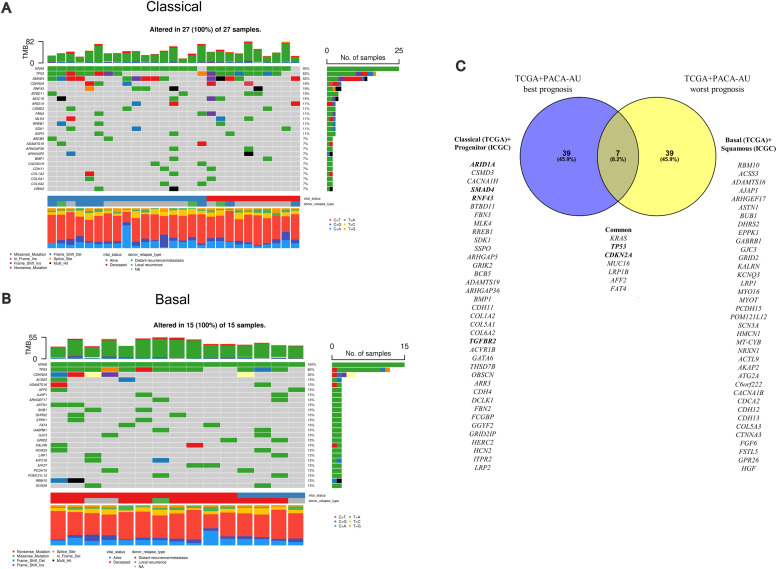
**Transcriptomic stratification in PDAC reveals subtype-specific somatic variants.** Oncoplots* of the top 25 most frequently mutated genes for consensus **(A)***n* = 27 *classical*-type and **(B)***n* = 16 *basal*-type PDAC cases (TCGA). **(C)** Overlap^ between the somatically mutated genes associated with best/worst prognosis subtypes across TCGA and ICGC PACA-AU cohorts combined. Genes highlighted in **bold** contain Tier 1 predicted oncogenic driver variants that have associated pharmacological inhibitors or chemotherapies (see Supplementary Table S1). *Mutated genes are ranked in order of the *total* number of *mutations* in each given gene (where genes may have >1 mutation present; black ‘multi-hit’), while the percentage to the right of each bar reflects the proportion of *samples* altered in the cohort. ^Created in Venny 2.1.

Comparisons between the genomic characteristics of each TCGA subtype showed different gene sets mutated in *classical* and *basal* subtypes (Fig. 3A, B). This was also true for PACA-AU prognostic subtypes (progenitor vs squamous) (Supplementary Figure 1A, B), but with little consensus between the two cohorts (Supplementary Figure 1C). To more reliably identify subtype-specific genetic variations robust to inevitable inter-study variability (e.g. tissue heterogeneity; WES vs WGS[27]), we considered the *union* of the top 25 most frequently mutated genes between similar prognostic groups for the two largest datasets (TCGA+ICGC). This revealed a handful of common genes detected at >10 % prevalence (*KRAS, TP53, CDKN2A, MUC16, LRP1B, AFF2, FAT4*), but with most genes/variants being subtype-specific (Fig. 3C), consistent with (1) the early acquisition of these common alterations during PDAC tumour development and (2) the molecular heterogeneity of PDAC tumours[24].

### *Identifying treatment biomarkers*

*In silico* functional analysis of *all* patient-specific somatic variants identified, using the most recent, freely available embedded Cancer Genome Interpreter (CGI)[28] analytic tool, identified numerous biomarkers of response/resistance to existing clinical treatments and/or pharmacological inhibitors (Supplementary Table S2) in various cancer contexts, including multiple variants in *KRAS, TP53, CDKN2A* and *LRP1B*.

Although *KRAS* is extensively mutated across both prognostic groups, *KRAS* p.G12C driver variants were unique to the best prognosis subtype tumours (Supplementary Table S2). This variant, rare in PDAC (<1 % patients)[29], has been shown to preferentially drive the RAF/RAL pathway, while the more common *KRAS*^G12D^ mutation (∼30 % PDAC patients) favours the PI3K/AKT pathway[29,30]. Targeted *KRAS*^G12C^ inhibitors sotorasib and adagrasib have recently received FDA approval for treatment of pre-treated mNSCLC [31,32] and have been shown to be safe and effective in treatment of advanced mPDAC, in a Phase I/II trial in *n* = 38 patients[33]. However, there is limited evidence to support the clinical use of *KRAS*^G12C^ inhibitors as effective monotherapies in *KRAS*^G12C^-mutated cancers, including PDAC, largely due to primary and acquired resistance [34]. So, while these developments have demonstrated *KRAS* as a therapeutically targetable oncogene generally, the targeting of *KRAS*^G12C^ in PDAC requires refining before it can be an effective mutation-specific therapeutic strategy for the ∼2 % PDAC patients with this *KRAS* variant.

Additionally, certain variants in *CDKN2A* (L104V, E120*, R58*, R80*) have been linked with treatment resistance to PD1 inhibitors, and treatment response to CDK4/6 inhibitors in cutaneous melanoma (CM; Supplementary Table S2), highlighting the usefulness of PGPA in identifying potentially clinically relevant patient- and cancer-specific therapeutic targets.

Of the somatic variants identified, CGI oncogenic classifications (bioactivity) (Supplementary Table S1), revealed several to be TIER 1 predicted driver variants, i.e. the gene activity is confirmed relevant to cancer, with mutations identified effecting oncogenic transformation. Only one was identified in the poorest outcome patient group – a splice donor variant in central cell-cycle regulator gene *ATM*, and linked with response to cisplatin chemotherapy, PARPi by olaparib, and PD1/PD-L1 inhibition in other solid tumours (Supplementary Table S2). However, this variant was uncommon in the sample set (1/16). Conversely, several TIER 1 oncogenic driver variants were identified in the best prognosis patient group, with some associated with response/resistance to specific drugs in other solid tumours, suggesting possible utility in pancreatic cancer: *ARID1A* (p.E1542*, p.Q1277*; responsive to EZH2, PD1, ATR & PARP inhibitors), *RNF43* (W159*, responsive to porcupine inhibitor) and *TP53* (S166*, Y205S, D259V, M246R, S241F, L194H, N131I; resistance to CDK4/6 inhibitor abemaciclib, cisplatin, MDM2 inhibitor; responsive to ATR inhibitor AZD6738, doxorubicin, decitabine (Dacogen), gemcitabine, mitomycin C).

Results of the above detailed output are also summarised in alluvial plots, showing clinically-actionable targets present in selectable proportions of the filtered dataset (5 %−25 %) and their responsiveness/resistance to available drugs (Supplementary Figure 2A, B), as well as a visual summary of the number of druggable gene categories represented in the selected dataset (Supplementary Figure 2C, D), that shows different clinically actionable genome targets between the two prognostic groups. While the most commonly mutated genes are common between prognostic subtypes (≥25 % of patients from both groups contain similar *KRAS* and *TP53* variants), distinct biomarker/drug combinations are apparent when less common variations are considered: only one other gene in the poor prognosis group was identified as harbouring variants linked to (among others) responsiveness to small molecule AURKA-VEGF inhibitor ilorasertib (CDKN2A R58*), whereas the better prognosis subgroup is associated with 6 additional gene/biomarker candidates (*RNF43, PIK3CA, ERCC4, CTNNB1, CDKN2A, ARID1A*), that have shown promise in treating other solid tumours. Additionally, by exploring the available CCLE database of 60 pancreatic cell lines, *in vitro* models with/without *KRAS* and/or *TP53* variants may be identified to support downstream functional studies.

These results demonstrate the value of PGPA in contextualising individual patient genetic profiles in suggesting possible treatment options, or refining research areas to pursue for more effective, stratified approaches in pancreatic cancer. We realise that these findings are based on a small set of stringently filtered samples between TCGA and PACA-AU datasets, considering the best vs worst outcome patients that may not reflect the complexity of distinguishing these patients in a typical cohort. For example, although the two main transcriptomic subtypes distinguishing good/poor outcome in PDAC are broadly *classical/progenitor* and *basal-like/squamous* [24,25], the poorest prognosis subtypes are under-represented in Collisson et al. (2011)(QM; 23 %) and Bailey et al. (2016)(basal; 21 %), compared to their equivalents in Moffitt et al. (2015) (basal; 43 %), which may introduce some bias in the final analysis.

Even so, this analysis of evolutionary trajectories in PDAC has revealed patterns that are increasingly recognised as key to treatment resistance and failure. For example, unstable genomic profiles enriched for DNA damage repair defects initially confer sensitivity to platinum agents or PARPi, but frequently develop resistance through restoration of homologous recombination, metabolic rewiring or PARP1 downregulation, consistent with recent findings in *BRCA2*-mutant PDAC [35,36]. Single cell RNAseq analyses have also revealed the coexistence of basal-like and classical phenotypes within the same tumours, with therapy-induced changes in the tumour microenvironment reducing responsiveness to immune checkpoint blockade [37]. Furthermore, tumour evolutionary timing analyses suggest that subtype-defining features may be intrinsic from early tumour development, rather than only acquired later [38], supporting the idea that stratification of patients by molecular subtype and/or genomic feature (e.g. in PGPA), could inform treatment strategies before resistance emerges. Together, these insights underscore the translational potential of PGPA to link evolutionary trajectories with resistance mechanisms and to guide future biomarker-driven therapeutic approaches.

### *Characterising patterns of gene expression in best- and worst-outcome tumours*

**Fig. 4:**
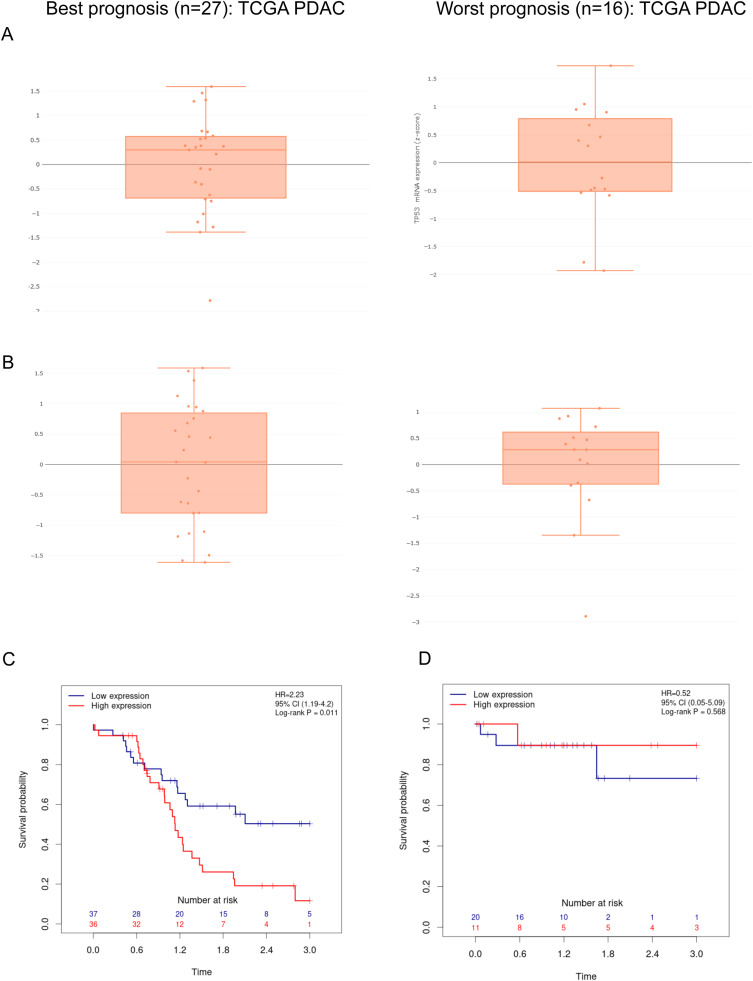
**Differentially expressed genes between classical/progenitor and basal-like/QM/squamous TCGA PDAC tumours.** Box plots showing the trends of **(A)** TP53 and **(B)** MUC16 mRNA expression levels across all patients in each filtered TCGA PDAC group; best prognosis (*n* = 27; classical/progenitor; left) and worst prognosis (*n* = 16; basal/squamous/QM; right). **C**. Kaplan-Meier curve showing elevated MUC16 expression significantly associated with lower patient survival over 3 years, from *n* = 402 PACA-AU PDAC patients with expression and outcome data (logrank *p* = 0.011; hazard ratio (HR)=2.23). **D.** No association between MUC16 mRNA expression levels and outcome were observed in *n* = 65 neuroendocrine carcinomas (PAEN-AU).

Inspecting the top 250 differentially expressed genes in both TCGA and ICGC filtered patient subgroups confirms scant overlap between genes differentially expressed in best and worst-outcome PDAC tumours. However, *classical/progenitor* tumours appear to have higher *TP53* expression compared to *QM/basal/squamous* tumours, consistent with its role as tumour suppressor (Fig. 4A) and mirrored in ICGC *progenitor* and *squamous* patient subgroups[19] (Supplementary Figure 3). Subtype-specific differences in expression were also observed for *MUC16* (encoding CA125 membrane glycoprotein) which was over-expressed in *QM/basal/squamous* subtype tumours, compared to *classical/progenitor* cases (Fig. 4B). Considering all *n* = 402 confirmed PDAC in PACA-AU (the largest single transcriptomic dataset), PGPA shows that *MUC16* over-expression is associated with significantly reduced patient survival (logrank *p* = 0.011; HR=2.23) (Fig. 4C), where no association was found for neuroendocrine tumours (Fig. 4D). Elevated CA125 has also recently been shown to be an independent prognostic marker of significantly shorter survival in *n* = 207 resectable PDAC patients, both before and after treatment[39]. Functionally, CA125 over-expression has been shown to promote tumourigenesis *in vitro* and *in vivo*[40,41], and monoclonal antibody mAb AR9.6 has recently shown potential as a specific and effective inhibitor of CA125 and its oncogenic effects in pancreatic and ovarian cancers[41,42].

### *Identifying clinically actionable genomic alterations in KRAS wild-type PDAC tumours*

Several studies have explored the genetic landscape of *KRAS* wild-type tumours, delineating several alterations that occur frequently in the absence of mutant *KRAS* [24,[43], [44], [45]]. In addition, a significant enrichment for somatic aberrations that target the RAS-MAPK pathway, either upstream or downstream of *KRAS*, has been observed in up to one-third of *KRAS* wild-type tumours[24,45],where *BRAF* alterations were prevalent and mutually exclusive with *KRAS* mutations[45]. However, alterations within genes that are not typically associated with *RAS* signalling have also been widely identified across *KRAS* wild-type PDAC tumours, and require further investigation to determine functional relevance[19,24].

### *Alternative oncogenic drivers amongst KRAS wild-type PDAC tumours*

**Fig. 5:**
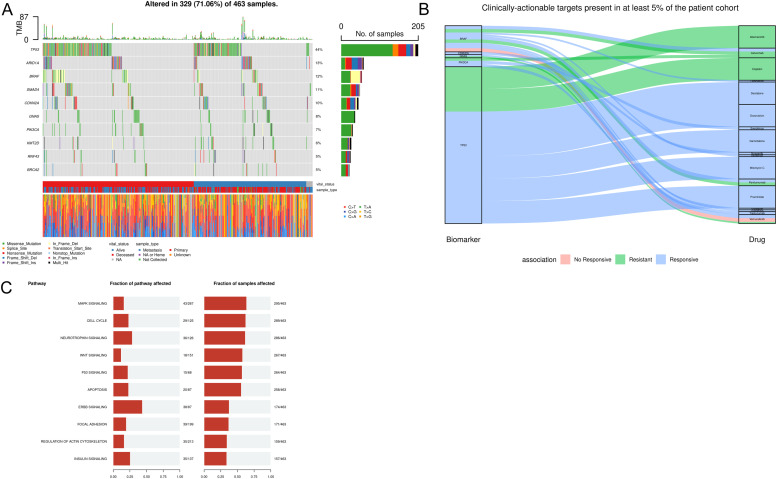
**Frequently altered genes and biological pathways amongst *n*=756 *KRAS* wild-type PDAC tumours from GENIE. (A)** Oncoplot showing the top 10 most frequently mutated genes in *KRAS* wild-type PDAC tumours (confirmed somatic missense mutations filtered out; insertions or duplications may still be present). **(B)** Alluvial plot showing gene targets harbouring any variants with therapeutic biomarker potential in ≥5 % of patients, as identified by the Cancer Genome Interpreter and based on data from OncoKB, CIVic (Clinical Interpretation of Variants in Cancer) and the Cancer Biomarkers database. **(C)** Altered biological pathways amongst *KRAS* wild-type PDAC tumours include MAPK and p53 signalling, as derived from the KEGG pathway database. The proportion of genes mutated in each pathway (left) and the proportion of all KRAS wild-type patients affected (x-axis) are given.

We used GENIE[12] as the largest available resource to identify *n* = 756 *KRAS* wild-type PDAC samples. To identify other likely molecular drivers in these tumours, predictions from CGI were analysed to evaluate the distribution of altered genes and their associated pathways. Mutations were detected across several genes previously reported to be altered in *KRAS* wild-type PDAC cases, including *TP53* (mutated in >40 % of the samples), *GNAS* and *BRAF*[46] (Fig. 5A). *In silico* biomarker predictions also showed that *BRAF, CDKN2A, NRAS, PIK3CA and TP53* variants demonstrated therapeutic potential in response to PARP, tyrosine kinase and VEGF inhibitors, immunotherapies and several chemotherapies (Fig. 5B).

The analysis of altered signalling pathways amongst mutated genes across *KRAS* wild-type samples revealed frequent alterations in pathways associated with MAPK signalling, P53 signalling, neurotrophin, cell cycle, wnt and apoptosis signalling, consistent with previous characterisations of core biological pathways involved in PDAC development and progression[19,20,24,40,46]. (Fig. 5C). These findings highlight the utility of PGPA for the prioritisation of functionally and biologically relevant variants amongst subgroups of PDAC tumours, with important implications for the characterisation of distinct molecular pathologies and the identification of novel therapeutic opportunities. A more detailed exemplar of this analysis of GENIE data in PGPA Analytics Hub is available in the website’s User Guide.

Using the PGPA Analytics Hub, we demonstrate how our integrated high-performance visualisation and analysis tool can be used to investigate the link between genomic and transcriptomic features and phenotypes of pancreatic cancer, providing an important step in defining potential subtype-specific therapeutic vulnerabilities.

## The PCRFTB data return module

The vision of precision medicine has driven unprecedented interest into biomarker-based studies (genomics, transcriptomics, proteomics) for pancreatic cancer, which are being adopted across research and development from early discovery through to clinical research and trials[47]. Fundamental to biomarker research is access to quality biospecimens and samples that have been well annotated with clinical and molecular data[7]. Whilst many biobanks have invested heavily in the IT infrastructure of sample management, most platforms are facing challenges in the effective sharing of returned data to drive investigative research across the pancreatic research community. A major challenge is the rise of so-called ‘big data’ from e.g. NGS and images that need to be integrated with large quantities of primary/secondary care information and other real-world healthcare data. Traditional biobanks are not usually set up to leverage these innovations, which have the potential to improve patient outcomes and accelerate the development and delivery of new therapies. PGPA is now the primary bioinformatics platform of the PCRFTB, providing a unique integrated resource of biological materials and associated clinical, molecular and radiological/imaging data.

In addition to providing a direct link to sample requests from the PCRFTB, the newly incorporated data return module in PGPA hosts clinical and molecular data returned to the biobank from studies undertaken using PCRFTB specimens, where published findings have been made available for researchers to review and analyse prior to submitting a tissue request. Studies are categorised according to the type of -omics data generated for each project (i.e., genomic, transcriptomic, proteomic), with alternative data types (e.g., summaries of staining or imaging results generated from experimental investigations) classified “other”, to simplify use. These classifications are presented in a summary table, which also provides a description of each project and details the different sample types (i.e., blood, tissues, cell lines) and cohort sizes used for each project. Users also have the option view this information as clinical summary plots for each individual cohort, prior to exploring available molecular data from each study.

Like other tissue repositories[48,49], PCRFTB has implemented a data return policy, where anonymised data derived from banked samples is returned to the tissue bank on completion of the study and made freely available to the research community, regardless of whether the study is ultimately published[7]. As associated sample datasets develop, more in-depth integrative analyses will be possible. Under *PCRFTB Data*, PGPA lists the details of any returned data and associated sample characteristics available for analysis, while *PCRFTB Research Projects* links directly to the relevant published report. So far, this has further enriched the data available for banked tissue sample and/or patients, and currently includes data on stromal[50], urinary miRNA[51], metallomic[52] and volatile organic compound[53] biomarkers, proteomics (ELISA)[54], circulating tumour cells (CTCs) and xenotransplantation models[55,56], germline and somatic mutations[57], a phase I clinical trial[58], and risk[59] and recurrence[60] predictions incorporating electronic health record data. To date, PCRFTB has processed 70 Expressions of Interest, supported 51 research projects and 20 peer-reviewed publications. Ultimately, each study contributes to the development of a ‘digital fingerprint’ for each patient, linking multi-modal data with longitudinal clinical information.

PGPA Analytics Hub is the web-based portal through which this enriched dataset can be accessed and compared with large scale pancreatic -omics data, with the unique benefit of also providing access to additional patient samples (*via* PCRFTB) for subsequent validation of molecular alterations with clinical potential. Here, we demonstrate the added value of tissue banking to precision cancer medicine, to translate research findings into prognostic and therapeutic tools using well-annotated curated tissues and associated clinical data.

## Discussion

A rapid expansion in high-throughput genomic and transcriptomic profiling of pancreatic diseases necessitates the development of sophisticated yet user-friendly analytics hubs to host the growing compendium of molecular and clinical datasets and enable integrated mining and analysis of available results. PGPA supports a range of data modalities to enable users across the diverse international pancreatic research community to identify and investigate trends in molecular data across disparate cohorts of patients, samples and cell lines easily and effectively. PGPA also provides an unprecedented opportunity to characterise distinct variations in tumour mutation landscapes, gene expression profiles and derived immunotypes that are associated with prognostic molecular subtypes. Candidate tumour drivers and biomarkers predictive of response to existing and novel clinical treatments can be identified and visualized, allowing suitable targets for downstream validation and pharmacological testing to be prioritised without the need for laborious data retrieval or processing tasks. Furthermore, PGPA is the gateway to a national tissue bank repository of >125 000 samples from >3980 donors and a growing repository of digitised radiological and H&E images, that researchers can access independently and apply for donor samples that match with their research question. The selection of samples is significantly improved by the availability of high-quality clinical data, curated and maintained by ISO-accredited PCRFTB.

Pancreas Genome Phenome Atlas is also the bioinformatics platform of the PCRFTB, pioneering a new generation in biobanking to support effective data sharing and promote collaborative studies, democratizing access to complex cancer genomics. By harmonising PCRFTB samples with clinical and molecular information from datasets returned to the biobank, PGPA provides an essential platform to support translational pancreatic research and fuel discoveries that can manifest clinically meaningful benefits for patients.

Practically, PGPA provides a pipeline from discovery to clinical application by enabling candidate biomarkers identified *in silico* to be validated using both public datasets and PCRFTB biospecimens, followed by assay development in cell lines and organoids, and even incorporation into prospective clinical studies. This integrated framework links computational analysis, experimental validation, and clinical annotation, thus supporting the translation of molecular findings into decision-making tools for prognosis, patient stratification, and therapy selection.

PCRFTB recently launched internationally (https://www.pcrf.org.uk/news/tissue-bank-launches-internationally/), improving opportunities for high-quality research into earlier diagnosis and treatment of pancreatic cancer. As -omics driven research continues to drive efforts to advance the characterisation of pancreatic diseases, PGPA supports the ongoing data analysis, integration and visualisation needs of the growing research community.

## Availability of data

PGPA uses publicly available datasets from TCGA (https://doi.org/10.1016/j.ccell.2017.07.007, https://doi.org/10.1016/j.immuni.2018.03.023), GENIE https://doi.org/10.1158/2159–8290.CD-17–0151), ICGC (https://doi.org/10.1093/database/bar026, https://doi.org/10.1038/s41587–019–0055–9 and CCLE (https://doi.org/10.1038/nature11003).

## Funding

The PCRFTB is funded by a Pancreatic Cancer Research Programme Grant to HMK and CC. This work was supported by Barts Charity (MGU0504) to CC. We also acknowledge the support of the National Institute for Health and Care Research Barts Biomedical Research Centre (NIHR203330); a delivery partnership of the Precision Medicine programme across Barts Health NHS Trust, Queen Mary University of London, St George’s University Hospitals NHS Foundation Trust and St George’s University of London. The funding bodies had no role in the design, collection, analysis or interpretation of data in this study, or the writing of the manuscript.

## Ethics approval

All donors provide written, informed consent, and all samples are collected, processed and stored at each of the participating centres (Barts, Leicester, Swansea, Oxford, Royal Free (London), Southampton, Newcastle, Plymouth, The London Clinic) under one Research Ethics Committee reference (13/SC/0593, renewed 18/SC/0629, renewed 23/SC/0282).

## CRediT authorship contribution statement

**Jorge Oscanoa:** Visualization, Software, Resources, Methodology. **Helen Ross-Adams:** Writing – review & editing, Writing – original draft, Validation, Project administration, Investigation, Formal analysis, Data curation. **Abu Z.M. Dayem Ullah:** Software, Resources, Data curation. **Trupti S. Kolvekar:** Visualization, Methodology, Data curation. **Lavanya Sivapalan:** Writing – original draft, Investigation, Formal analysis. **Emanuela Gadaleta:** Visualization, Resources. **Graeme J. Thorn:** Visualization, Software. **Maryam Abdollahyan:** Resources, Data curation. **Ahmet Imrali:** Resources, Data curation. **Amina Saad:** Resources, Data curation. **Rhiannon Roberts:** Resources, Project administration, Data curation. **Christine S. Hughes:** Resources, Project administration, Data curation. **PCRFTB, Hemant M. Kocher:** Writing – review & editing, Supervision, Resources, Funding acquisition, Conceptualization. **Claude Chelala:** Writing – review & editing, Supervision, Project administration, Funding acquisition, Data curation, Conceptualization.

## Declaration of competing interest

The authors declare that they have no known competing financial interests or personal relationships that could have appeared to influence the work reported in this paper.

## Appendix Supplementary materials

Image, application 1

Image, application 2

Image, application 3

Image, application 4

Image, application 5

## Footnotes

Supplementary material associated with this article can be found, in the online version, at doi:10.1016/j.tranon.2025.102550.

## References

[1] Siegel R.L., Miller K.D., Jemal A. (2020). Cancer statistics, 2020. CA Cancer J. Clin..

[2] Pereira S.P., Oldfield L., Ney A., Hart P.A., Keane M.G., Pandol S.J. (2020). Early detection of pancreatic cancer. Lancet Gastroenterol. Hepatol..

[3] Abboud Y., Samaan J.S., Oh J., Jiang Y., Randhawa N., Lew D. (2023). Increasing pancreatic cancer incidence in young women in the. Gastroenterology.

[4] Brezgyte G., Shah V., Jach D., Crnogorac-jurcevic T. (2021). Non-invasive biomarkers for earlier detection of pancreatic cancer—a comprehensive review. Cancers (Basel).

[5] Marzec J., Dayem Ullah A.Z., Pirrò S., Gadaleta E., Crnogorac-Jurcevic T., Lemoine N.R. (2018). The pancreatic expression database: 2018 update. NAR (Database Issue).

[6] Oscanoa J., Sivapalan L., Gadaleta E., Dayem Ullah A.Z., Lemoine N.R., Chelala C. (2020). SNPnexus: a web server for functional annotation of human genome sequence variation (2020 update). Nucleic Acids Res..

[7] Balarajah V., Ambily A., Dayem Ullah A.Z., Imrali A., Dowe T., Al-Sarireh B. (2016). Pancreatic cancer tissue banks: where are we heading?. Future Oncol..

[8] Imrali A., Hughes C.S., Coetzee A.S., Delvecchio F.R., Saad A., Roberts R. (2020). Validation of a novel, flash-freezing method: aluminum platform. Curr. Protoc. Essent. Lab. Tech..

[9] Pancreatic Cancer Research Fund, PCRF tissue bank achieves landmark international accreditation. https://www.pcrf.org.uk/news/pcrf-tissue-bank-achieves-landmark-international-accreditation/. (Accessed 8 September 2025).

[10] Weinstein J.N., Collisson E.A., Mills G.B., Shaw K.R.M.M., Ozenberger B.A., Ellrott K. (2013). The cancer genome atlas pan-cancer analysis project. Nat. Genet..

[11] Barretina J., Caponigro G., Stransky N., Venkatesan K., Margolin A.A., Kim S. (2012). The cancer cell line encyclopedia enables predictive modelling of anticancer drug sensitivity. Nature.

[12] Sweeney S.M., Cerami E., Baras A., Pugh T.J., Schultz N., Stricker T. (2017). AACR project genie: powering precision medicine through an international consortium. Cancer Discov..

[13] Zhang J., Baran J., Cros A., Guberman J.M., Haider S., Hsu J. (2011). International cancer genome consortium data portal–a one-stop shop for cancer genomics data. Database (Oxf.).

[14] Zhang J., Bajari R., Andric D., Gerthoffert F., Lepsa A., Nahal-Bose H. (2019). The international cancer genome consortium data portal. Nat. Biotechnol..

[15] Peran I., Madhavan S., Byers S.W., McCoy M.D. (2018). Curation of the pancreatic ductal adenocarcinoma subset of the cancer genome atlas is essential for accurate conclusions about survival-related molecular mechanisms. Clin. Cancer Res..

[16] Nicolle R., Raffenne J., Paradis V., Couvelard A., de Reynies A., Blum Y. (2019). Prognostic biomarkers in pancreatic cancer: avoiding errata when using the TCGA dataset. Cancers (Basel).

[17] Collisson E.A., Sadanandam A., Olson P., Gibb W.J., Truitt M., Gu S. (2011). Subtypes of pancreatic ductal adenocarcinoma and their differing responses to therapy. Nat. Med..

[18] Moffitt R.A., Marayati R., Flate E.L., Volmar K.E., Loeza S.G.H., Hoadley K.A. (2015). Virtual microdissection identifies distinct tumor- and stroma-specific subtypes of pancreatic ductal adenocarcinoma. Nat. Genet..

[19] Bailey P., Chang D.K., Nones K., Johns A.L., Patch A.M., Gingras M.C. (2016). Genomic analyses identify molecular subtypes of pancreatic cancer. Nature.

[20] Waddell N., Pajic M., Patch A.M., Chang D.K., Kassahn K.S., Bailey P. (2015). Whole genomes redefine the mutational landscape of pancreatic cancer. Nature.

[21] de Santiago I., Yau C., Heij L., Middleton M.R., Markowetz F., Grabsch H.I. (2019). Immunophenotypes of pancreatic ductal adenocarcinoma: meta-analysis of transcriptional subtypes. Int. J. Cancer.

[22] Thorsson V., Gibbs D.L., Brown S.D., Wolf D., Bortone D.S., Ou Yang T.-H. (2018). The immune landscape of cancer. Immunity.

[23] Saillard C., Delecourt F., Schmauch B., Moindrot O., Svrcek M., Bardier-Dupas A. (2023). Pacpaint: a histology-based deep learning model uncovers the extensive intratumor molecular heterogeneity of pancreatic adenocarcinoma. Nat. Commun..

[24] Raphael B.J., Hruban R.H., Aguirre A.J., Moffitt R.A., Yeh J.J., Stewart C. (2017). Integrated genomic characterization of pancreatic ductal adenocarcinoma. Cancer Cell.

[25] Sinkala M., Mulder N., Martin D. (2020). Machine learning and network analyses reveal disease subtypes of pancreatic cancer and their molecular characteristics. Sci. Rep..

[26] Sivakumar S., de Santiago I., Chlon L., Markowetz F. (2017). Master regulators of oncogenic KRAS response in pancreatic cancer: an integrative network biology analysis. PLoS Med..

[27] Ellrott K., Bailey M.H., Saksena G., Covington K.R., Kandoth C., Stewart C. (2018). Scalable open science approach for mutation calling of tumor exomes using multiple genomic pipelines. Cell Syst..

[28] Tamborero D., Rubio-Perez C., Deu-Pons J., Schroeder M.P., Vivancos A., Rovira A. (2018). Cancer genome interpreter annotates the biological and clinical relevance of tumor alterations. Genome Med..

[29] Kwan A.K., Piazza G.A., Keeton A.B., Leite C.A. (2022). The path to the clinic: a comprehensive review on direct KRASG12C inhibitors. J. Exp. Clin. Cancer Res..

[30] Ihle N.T., Byers L.A., Kim E.S., Saintigny P., Lee J.J., Blumenschein G.R. (2012). Effect of KRAS oncogene substitutions on protein behavior: implications for signaling and clinical outcome. J. Natl. Cancer Inst..

[31] Nakajima E.C., Drezner N., Li X., Mishra-Kalyani P.S., Liu Y., Zhao H. (2022). FDA approval summary: sotorasib for KRAS G12C-mutated metastatic NSCLC. Clin. Cancer Res..

[32] Jänne P.A., Riely G.J., Gadgeel S.M., Heist R.S., Ou S.-H.I., Pacheco J.M. (2022). Adagrasib in non–small-cell lung cancer harboring a KRAS G12C mutation. N. Engl. J. Med..

[33] Strickler J.H., Satake H., George T.J., Yaeger R., Hollebecque A., Garrido-Laguna I. (2023). Sotorasib in KRAS p.G12C–mutated advanced pancreatic cancer. N. Engl. J. Med..

[34] Miyashita H., Kato S., Hong D.S. (2024). KRAS G12C inhibitor combination therapies: current evidence and challenge. Front. Oncol..

[35] Stoof J., Andrieu C., O’connell F., O’sullivan J., Lowery M.A., Walsh N. (2025). Mechanisms of resistance to PARPi in pancreatic ductal adenocarcinoma. J. Cell. Mol. Med..

[36] Zou Y., Zhang H., Chen P., Tang J., Yang S., Nicot C. (2025). Clinical approaches to overcome PARP inhibitor resistance. Mol. Cancer.

[37] Werba G., Weissinger D., Kawaler E.A., Zhao E., Kalfakakou D., Dhara S. (2023). Single-cell RNA sequencing reveals the effects of chemotherapy on human pancreatic adenocarcinoma and its tumor microenvironment. Nat. Commun..

[38] Mullen K.M., Hong J., Attiyeh M.A., Hayashi A., Sakamoto H., Kohutek Z.A. (2025). The evolutionary forest of pancreatic cancer. Cancer Discov..

[39] Napoli N., Kauffmann E.F., Ginesini M., Lami L., Lombardo C., Vistoli F. (2023). Ca 125 is an independent prognostic marker in resected pancreatic cancer of the head of the pancreas. Updates Surg..

[40] Qi Z.H., Xu H.X., Zhang S.R., Xu J.Z., Li S., Gao H.L. (2018). RIPK4/PEBP1 axis promotes pancreatic cancer cell migration and invasion by activating RAF1/MEK/ERK signaling. Int. J. Oncol..

[41] Thomas D., Sagar S., Liu X., Lee H.R., Grunkemeyer J.A., Grandgenett P.M. (2021). Isoforms of MUC16 activate oncogenic signaling through EGF receptors to enhance the progression of pancreatic cancer. Mol. Ther..

[42] Sharma S.K., Mack K.N., Piersigilli A., Pourat J., Edwards K.J., Keinänen O. (2022). ImmunoPET of ovarian and pancreatic cancer with AR9.6, a novel MUC16-targeted therapeutic antibody. Clin. Cancer Res..

[43] Heining C., Horak P., Uhrig S., Codo P.L., Klink B., Hutter B. (2018). NRG1 Fusions in KRAS wild-type pancreatic cancer. Cancer Discov..

[44] Luchini C., Paolino G., Mattiolo P., Piredda M.L., Cavaliere A., Gaule M. (2020). KRAS wild-type pancreatic ductal adenocarcinoma: molecular pathology and therapeutic opportunities. J. Exp. Clin. Cancer Res..

[45] Singhi A.D., George B., Greenbowe J.R., Chung J., Suh J., Maitra A. (2019). Real-time targeted genome profile analysis of pancreatic ductal adenocarcinomas identifies genetic alterations that might Be targeted with existing drugs or used as biomarkers. Gastroenterology.

[46] Philip P.A., Azar I., Xiu J., Hall M.J., Hendifar A.E., Lou E. (2022). Molecular characterization of KRAS wild-type tumors in patients with pancreatic adenocarcinoma. Clin. Cancer Res..

[47] Herbst B., Zheng L. (2019). Precision medicine in pancreatic cancer: treating every patient as an exception. Lancet Gastroenterol. Hepatol..

[48] Gadaleta E., Pirrò S., Dayem Ullah A.Z., Marzec J., Chelala C. (2018). BCNTB bioinformatics: the next evolutionary step in the bioinformatics of breast cancer tissue banking. Nucleic Acids Res..

[49] Speirs V. (2021). Quality considerations when using tissue samples for biomarker studies in cancer research. Biomark. Insights.

[50] Goulart M.R., Watt J., Siddiqui I., Lawlor R.T., Imrali A., Hughes C. (2021). Pentraxin 3 is a stromally-derived biomarker for detection of pancreatic ductal adenocarcinoma. NPJ Precis. Oncol..

[51] Debernardi S., Massat N.J., Radon T.P., Sangaralingam A., Banissi A., Ennis D.P. (2015). Noninvasive urinary miRNA biomarkers for early detection of pancreatic adenocarcinoma. Am. J. Cancer Res..

[52] Schilling K., Larner F., Saad A., Roberts R., Kocher H.M., Blyuss O. (2020). Urine metallomics signature as an indicator of pancreatic cancer. Metallomics.

[53] Daulton E., Wicaksono A.N., Tiele A., Kocher H.M., Debernardi S., Crnogorac-Jurcevic T. (2021). Volatile organic compounds (VOCs) for the non-invasive detection of pancreatic cancer from urine. Talanta.

[54] Debernardi S., O’Brien H., Algahmdi A.S., Malats N., Stewart G.D., Pljesa-Ercegovac M. (2020). A combination of urinary biomarker panel and PancRISK score for earlier detection of pancreatic cancer: a case-control study. PLoS Med..

[55] Raj D., Yang M.H., Rodgers D., Hampton E.N., Begum J., Mustafa A. (2019). Switchable CAR-T cells mediate remission in metastatic pancreatic ductal adenocarcinoma. Gut.

[56] Raj D., Nikolaidi M., Garces I., Lorizio D., Castro N.M., Caiafa S.G. (2021). CEACAM7 is an effective target for CAR T-cell therapy of pancreatic ductal adenocarcinoma. Clin. Cancer Res..

[57] Sivapalan L., Thorn G.J., Gadaleta E., Kocher H.M., Ross-Adams H., Chelala C. (2022). Longitudinal profiling of circulating tumour DNA for tracking tumour dynamics in pancreatic cancer. BMC Cancer.

[58] Kocher H.M., Basu B., Froeling F.E.M.M., Sarker D., Slater S., Carlin D. (2020). Phase I clinical trial repurposing all-trans retinoic acid as a stromal targeting agent for pancreatic cancer. Nat. Commun..

[59] Zardab M., Balarajah V., Banerjee A., Stasinos K., Saad A., Imrali A. (2023). Differentiating ductal adenocarcinoma of the pancreas from benign conditions using routine health records: a prospective case-control study. Cancers (Basel).

[60] Ang A., Michaelides A., Chelala C., Ullah D., Kocher H.M. (2024). Prognostication for recurrence patterns after curative resection for pancreatic ductal adenocarcinoma. Ann. Hepatobiliary Pancreat. Surg..

