# Supplementary material for: A central research portal for mining pancreatic clinical and molecular datasets and accessing biobanked samples
Source: Transl Oncol. 2025 Oct 3;62:102550. doi: 10.1016/j.tranon.2025.102550 (PMC12523802; doi:10.1016/j.tranon.2025.102550)
Supplement: Supplementary file 3 [file mmc3.pdf]

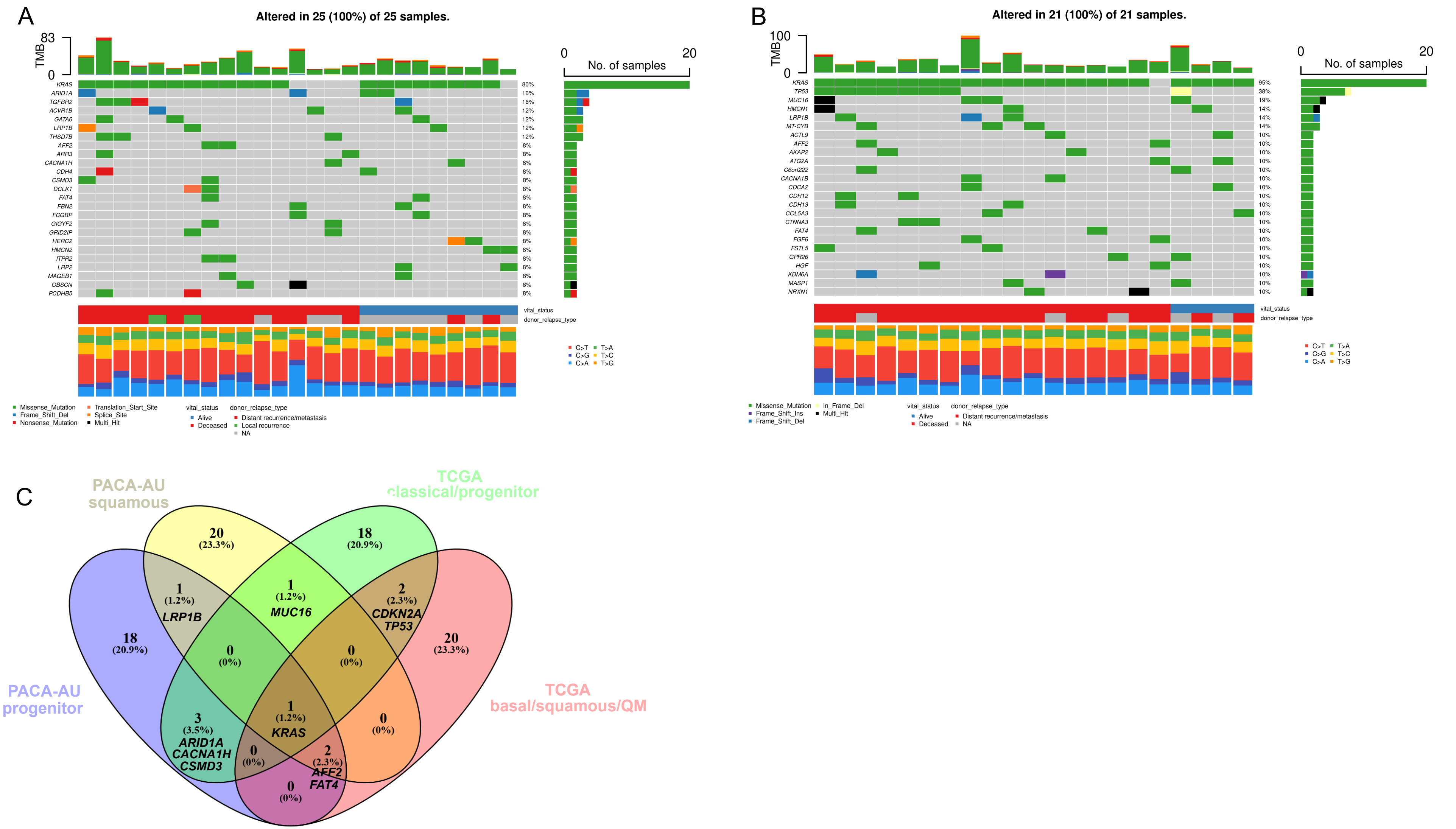

Supplementary Figure 1. Frequently mutated genes in ICGC PDAC (PACA-AU) Progenitor (best prognosis) and Squamous (worst prognosis) subtypes.

Oncoplots* showing the top 25 most frequently mutated genes in ICGC PACA-AU PDAC samples classified as either (A) progenitor (n=25) or (B) squamous (n=21) subtype. (C) Venn diagram showing limited overlap between somatic variants underlying the best/worst prognosis transcriptomic subtypes from the TCGA and ICGC cohorts separately: classical vs basal and progenitor vs. squamous, respectively. Plot created in Venny 2.1.

*Mutated genes are ranked in order of the total number of mutations in each given gene (genes with >1 mutation present are shown in black as ‘multi-hit’), while the percentage annotations to the right of each bar reflects the proportion of samples altered in the cohort.
