# Supplementary material for: A central research portal for mining pancreatic clinical and molecular datasets and accessing biobanked samples
Source: Transl Oncol. 2025 Oct 3;62:102550. doi: 10.1016/j.tranon.2025.102550 (PMC12523802; doi:10.1016/j.tranon.2025.102550)
Supplement: Supplementary file 4 [file mmc4.pdf]

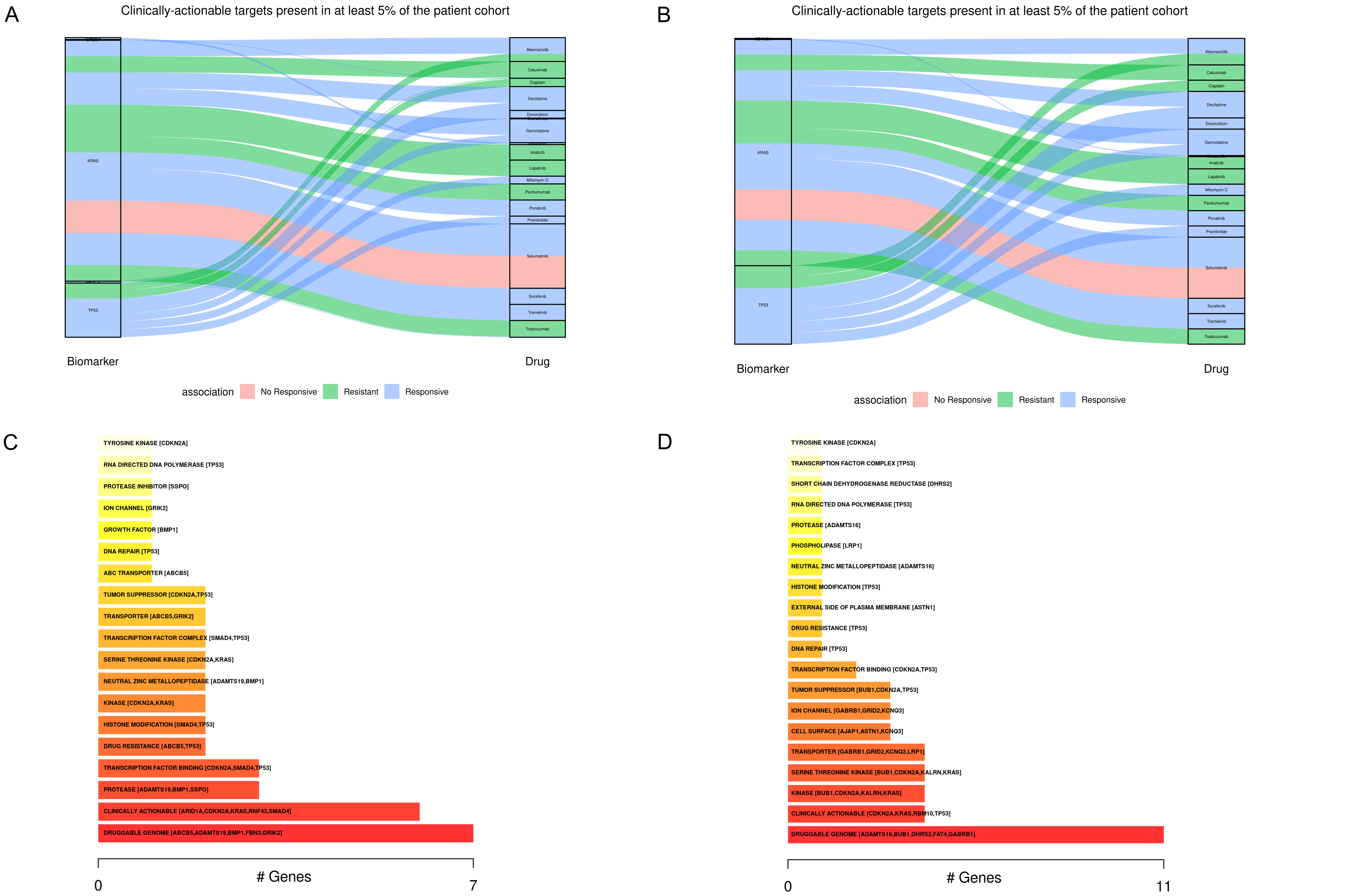

Supplementary Figure 2. Summary of the most frequently altered clinically actionable targets in best and worst prognosis TCGA PDAC tumours.

Alluvial plots showing the most frequently altered clinically actionable gene targets (KRAS and TP53) present in 5% of the selected patient cohort across (A) classical/progenitor/classical (best prognosis), and (B) basal-like/QM/squamous (worst prognosis) subtypes, as identified by the Cancer Genome Interpreter; subtype-specific gene/biomarker combinations not present at >5% are highlighted. The number and type of mutated genes associated with each patient group are further summarised into functional categories, compiled from the Drug Interaction Database: best prognosis (C) vs worst prognosis (D).
