# Supplementary material for: A central research portal for mining pancreatic clinical and molecular datasets and accessing biobanked samples
Source: Transl Oncol. 2025 Oct 3;62:102550. doi: 10.1016/j.tranon.2025.102550 (PMC12523802; doi:10.1016/j.tranon.2025.102550)
Supplement: Supplementary file 5 [file mmc5.pdf]

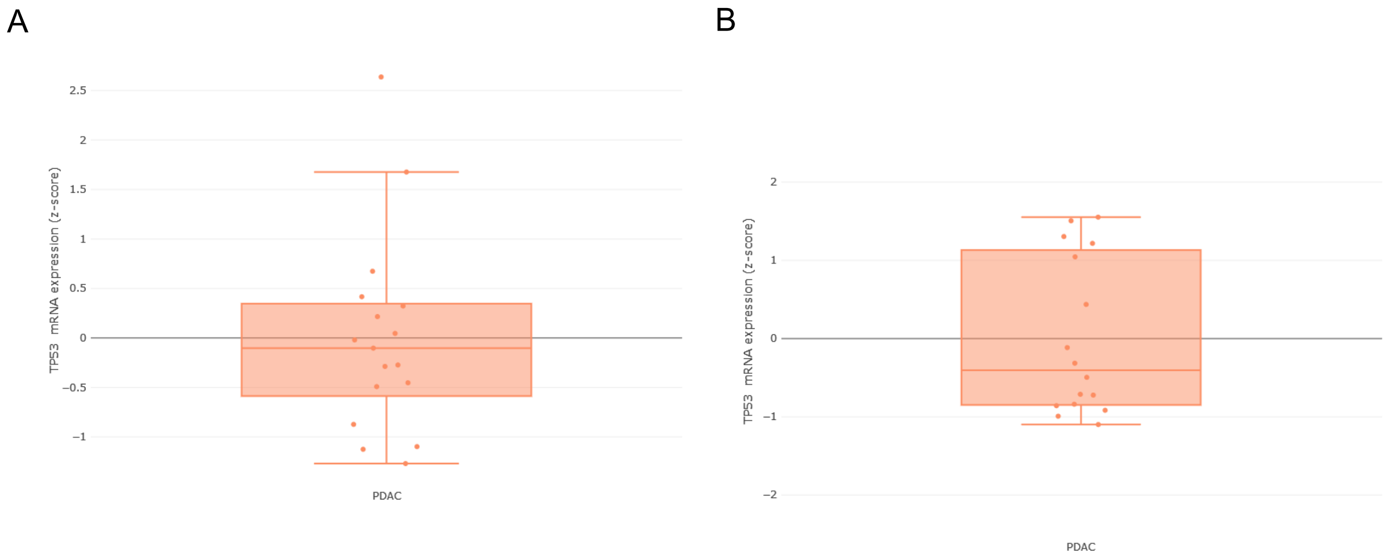

Supplementary Figure 3. mRNA expression levels of TP53 in ICGC PACA-AU patients.

Box and whisker plots showing the distribution of TP53 mRNA expression levels in the ICGC PACA-AU PDAC patients, filtered according to Bailey et al. (2016) prognostic subtype: (A) Progenitor subtype (best prognosis) has relatively higher median levels of TP53 expression than (B) Squamous subtype (worst prognosis).
